# High Expression of RhoF Predicts Worse Overall Survival: A Potential Therapeutic Target for non-M3 Acute Myeloid Leukemia

**DOI:** 10.7150/jca.52648

**Published:** 2021-07-25

**Authors:** Yue Hou, Jie Zi, Zheng Ge

**Affiliations:** Department of Hematology, Zhongda Hospital, Medical School of Southeast University, Institute of Hematology Southeast University, Nanjing 210009, China

**Keywords:** RhoF, acute myeloid leukemia, survival, clinical feature, therapeutic target

## Abstract

Rho GTPases are involved in multiple human malignancies and diverse biological functions. However, the patterns and prognostic significance of the expression of RhoD subfamily in acute myeloid leukemia (AML) remain unknown. Here, we detected the expressions of RhoD subfamily genes in AML on the basis of several published datasets and analyzed the survival of RhoD subfamily across the TCGA profiles and in a GEO series. We found that the expression of RhoF, but not RhoD, increased in AML patients in TCGA and GEO (all P<0.001); the survival analysis of two independent cohorts demonstrated that higher RhoF expression was significantly associated with poorer overall survival (OS) (P<0.001), whereas RhoD expression had no significant effect on OS in patients with AML (P>0.05); the subgroup analysis showed that high RhoF expression was correlated with poor 1-, 3-, and 5-year OS (P<0.05 for all); upregulated RhoF expression had a more significant prognostic value for OS in the younger patients (age<60), the intensive chemotherapy group, and wild-type groups (IDH1, NRAS, and TP53) (P<0.05 for all). Multivariate analysis indicated high RhoF expression as a strongly independent unfavorable prognostic factor for OS in patients without transplantation (P<0.05). Furthermore, a higher RhoF expression was closely associated with an older age, intermediate-/poor-risk cytogenetics and mutations in IDH1, NRAS, and TP53. RhoF expression was negatively correlated with BM blasts (P=0.020) and WBC (P=0.003). These findings suggest that high RhoF expression is associated with worsening OS in AML patients and is a potential therapeutic target for the treatment of AML.

## Introduction

Acute myeloid leukemia (AML) is the most common hematological malignancy and is characterized by the malignant clonal expansion of progenitor cells coupled by differentiation arrest 1. In recent decades, the incidence of AML has increased with the aging of the population 2. Although advances in the treatment of AML have been achieved in certain areas such as acute promyelocytic leukemia (APL), the overall survival (OS) of elderly patients (older than 65 years of age) remains low 3, 4. The prognosis of AML is determined by chromosomal abnormalities and fusion genes. With the advancement of microarray technology and next-generation sequencing, several new mutations have been identified in AML 5. However, clinical physicians are still faced with challenges posed by the lack of current knowledge of the molecular mechanisms underlying the occurrence, development, and inevitable recurrence of AML. Thus, it is highly urgent to identify reliable and practical prognostic biomarkers as novel AML treatment targets.

The Rho GTPase family consists of 8 subfamilies (RhoA, Rac, Cdc42, Rnd, RhoD, RhoBTB, RhoU and RhoH)^6^, ^7^. The most intensively studied members are RhoA, Rac and Cdc42, which have been demonstrated to be molecular switches that regulate actin organization, cell adhesion and migration 8. In addition, the Rho protein regulates quite a few other cell functions, including growth, apoptosis, cell cycle progression and genome stability 9. Available literature has documented controversial roles of Rho GTPase family, with some genes associated with pro-tumorigenic functions while others serving tumor-suppressing roles 10, 11, which complicates the role of these proteins in cancer. Previous studies provide evidence with respect to the pro-proliferation of normal and leukemic B cells by RhoA, Rac, RhoH and Rap GTPases. They contribute to both chemokine and BCR signaling pathways, acting predominantly through their effects on adhesion and cytoskeletal dynamics 12. In AML, the increased expression and activity of Cdc42 are associated with the transformation of HSCs/P into AML, which in turn blocks the differentiation of leukemia cells by controlling division symmetry 13. Nevertheless, it remains blurred how RhoA and Rac modulate the differentiation of AML cells 14, 15.

Small Rho GTPase Rif (RhoF), a member of the RhoD subfamily (RhoD and RhoF), is expressed in neuronal cells, hematopoietic cells and immune tissues, and promotes the development of murine B cells instead of T cells 16. Instead of the mediation of Cdc42 via mDia2, RhoF may independently induce filopodia 17. Moreover, RhoF-/- platelets that form filopodia and have normal actin dynamics are dispensable for the platelet function 18. RhoD affects the regulation of the intracellular transport of vesicles 19. Therefore, RhoF and RhoD have unique impacts as master regulators of membrane trafficking and the integration of cytoskeletal reorganization by triggering phenotypic alterations in cell behavior that distinguish them from classical Rho GTPases 20. Previous work has determined that malignant cells and tissues of B-cell-derived lymphoma originating from germinal center express higher levels of RhoF than their purified normal cell counterparts 21. These results suggest that the abnormal expression of the RhoD subfamily may have a latent and complex role in tumor promotion or suppression. However, few studies have focused on the mRNA expression of RhoD subfamily proteins in cancers and their association with clinical characteristics and prognoses. The clinical significance and exact role of the RhoD subfamily in AML remain blurred.

Therefore, it is of great clinical importance to evaluate the prognostic value of the RhoD subfamily in AML patients. To elucidate the potential relationship between RhoD subfamily expression and AML patient outcomes, we analyzed the mRNA expression features of the RhoD subfamily in the Cancer Cell Line Encyclopedia (CCLE), Gene Expression Profiling Interactive Analysis (GEPIA), Gene Expression Omnibus (GEO), and TCGA databases and conducted a survival analysis based on the cBioPortal TCGA profile.

## Materials and Methods

### Data resource and description

The expression of the Rho GTPase family in 16 AML cell lines were obtained from the EMBL-EBI dataset (https://www.ebi.ac.uk), which provides free access to numerous bioinformatics sequence analysis applications that contain gene expression characteristics in diseases and human cancer cell lines 22.

The mRNA expression of the Rho GTPase family in various cancers was compared with an online tool, CCLE (https://portals.broadinstitute.org/ccle), which accumulates massive gene expression and mutation data from human cancer cell lines 23.

**Figure 1:**
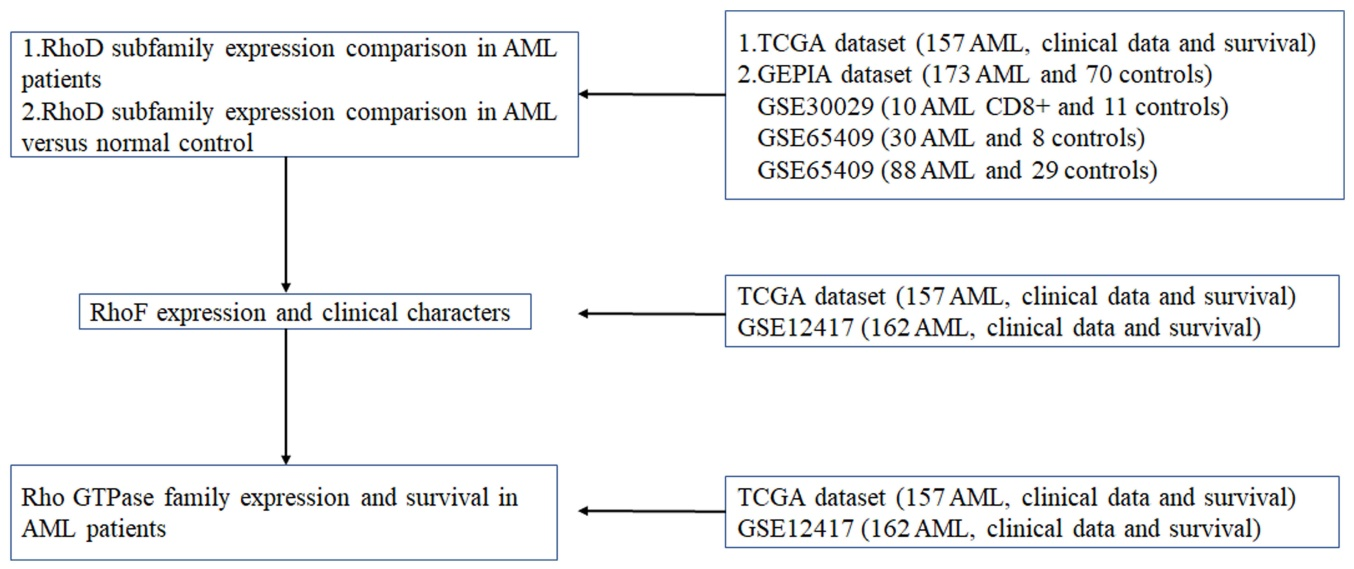
Flow chart for use of the datasets.

In this study, six datasets are downloaded and used for different purposes. Figure 1 is a flow chart showing all the used datasets and applications.

TCGA dataset: all the publicly available non-M3 AML RNA-Seq data and the clinical data of 157 of 200 newly diagnosed adult AML patients from the TCGA dataset (Acute Myeloid Leukemia, NEJM 2013)^24^ were downloaded from the cBioPortal dataset (http://www.cBioPortal.org/), which includes mutation conditions, median expression data, survival data and FAB and NCCN risk classification information. For FAB classification, 155 of 157 patients were classified into M0 to M7, and 2 patients were not classified in original data, which was indicated as “NA” in Table 2. For NCCN classification, 154 of 157 patients were classified to favorable, intermediate and poor, respectively, but 3 patients were not classified in original data, which was indicated as “NA” in Table 2.

GEPIA dataset: The RhoD subfamily expression levels in AML (n=173) and normal people (n=70) were determined using the GEPIA online platform. GEPIA, as a web-based tool provides customizable functions that include differential expression analysis, correlation analysis, similar gene detection, and patient survival analysis on the basis of TCGA and GTEx data 25.

Four GEO microarray series: In order to compare the expression of RhoF in AML versus normal control, three datasets were downloaded from the National Center for Biotechnology Information (NCBI) Gene Expression Omnibus (https://www.ncbi.nlm.nih.gov/geo/). There are GEO microarray series including GSE14924 (10 AML CD8+ and 11 controls, Affymetrix U133 Plus 2.0 Array), GSE65409 (30 AML and 8 controls, Illumina HumanHT-12 V3.0 beadchip) and GSE30029 (90 AML and 31 controls, Illumina HumanHT-12 V3.0 beadchip). In GSE30029, we deleted a maximum and a minimum respectively, which were far away from other data, resulting in a particularly large SD (88 AML and 29 controls). We also downloaded an independent cohort of 162 cytogenetically normal AML patients from GEO (GSE12417) to investigate the involvement of Rho GTPase family in survival.

This research mainly utilized the above datasets to conduct three major analyses.

First, TCGA dataset (Acute Myeloid Leukemia, NEJM 2013)^24^ was used to compare the expression of RhoD subfamily members in non-M3 AML patients. GEPIA dataset was used to compare the expression of RhoD subfamily members in AML patients versus normal control. In order to verify the expression level of RhoF in AML and normal control, we selected three independent cohorts including GSE14924, GSE65409 and GSE30029.

Second, we studied the relationship between the expression level of RhoF and the clinical characteristics of AML patients by TCGA dataset (Acute Myeloid Leukemia, NEJM 2013) 24 and GSE12417 because of the detailed and complete clinical data.

Third, only TCGA dataset (Acute Myeloid Leukemia, NEJM 2013) 24 and GSE12417 were attached with complete survival data, so they were used to do a series of survival analysis.

### Cell culture and qRT-PCR

To explore the mRNA expression of RhoF, AML cell lines (U937 and THP-1) were procured from ATCC (American Type Collection Center, Manassas, VA, USA). U937 and THP-1 cells were cultured in RPMI-1640 (Roswell Park Memorial Institute-1640, Gibco, Rockville, MD, USA) supplemented with 10% FBS (fetal bovine serum, Gibco, Rockville, MD, USA). The cells were grown at 37°C with 5% CO_2_ according to manufacturers' instructions.

Total RNA was extracted by TRIzol (Invitrogen, USA), and reverse transcription was conducted using PrimeScript™ RT Master Mix (Perfect Real Time, TaKaRa, Dalian, China) to obtain cDNA. Then, qRT-PCR was performed under the following conditions using TaKaRa SYBR Supermix (TaKaRa, Dalian, China) on a StepOne Plus analysis system (Applied Biosystems, Foster City, CA, USA): pre-denaturation at 95 °C for 30 s, pre-denaturation at 95 °C for 30 s, and annealing and extension at 60 °C for 34 s, for a total of 40 cycles. GAPDH was choosen as the internal reference, and the relative expression of RhoF was calculated by 2-ΔΔCt. The following primers were used in qRT-PCR: RhoF (F5'-AGCAAGGAGGTGACCCTGAAA-3', R 5'-CCGCAGCCGGTCATAGTC-3'); GAPDH (F5'-TTGGTATCGTGGAAGGACTCA-3', R5'-TGTCATCATATTTGGCAGGTTT-3').

### Statistical Analysis

After missing data were deleted, TCGA and GSE12417 cohort patients were divided into a high-expression group and a low-expression group with the median expression values of the Rho GTPase family as the cutoff points to clarify the connection between Rho GTPase family expression levels and clinical characteristics. All data were processed with IBM SPSS Statistics 26 and GraphPad Prism 8 software. The correlation between RhoF expression and clinical and laboratory parameters was analyzed by Spearman rank correlation coefficient tests, chi-square tests and Nonparametric tests. The difference in patient survival was analyzed by the Kaplan-Meier method and log-rank test with survival curves. Multivariate analysis adopted the Cox proportional hazards model. A P value of <0.05 was considered statistically significant.

### Identification of Co-expressed genes and RhoF-related genes

On GEPIA online platform, we screened 7214 differentially-expressed genes (DEGs) in AML and normal samples with a P value of <0.05 and logFC value of > 1 as the cutoff criteria using the RNA seq data. From the cBioPortal online platform, 1815 RhoF co-expressed genes were obtained in AML patients with the condition set at P<0.001. The intersection of DEGs and co-expressed genes was taken as RhoF-related genes according to WebTool Bioinformatics & Evolutionary Genomics (http://bioinformatics.psb.ugent.be/webtools/Venn/). 736 RhoF-related genes in AML patients were used for further bioinformatics analysis.

### KEGG, GO and GSEA analysis

The Database for Annotation, Visualization, and Integrated Discovery (DAVID; http://www.david.niaid.nih.gov) was accessed as a web-based tool to support Gene Ontology (GO) enrichment analysis and KEGG metabolic process analysis 26. A P-value of <0.05 was considered to indicate a significant difference. The results of the enrichment analysis were visualized in the bubble chart plotted by the R package “ggplot2” 27.

Gene Set Enrichment Analysis (GSEA) derives its power from focusing on gene sets and reveals many common biological pathways 28. RhoF was processed in GSEA to reveal potential biological pathways in AML with a nominal cutoff P value of < 0.05.

### Protein-protein interaction (PPI) network analysis

The STRING database (http://string-db.org) integrates a large quantity of data to predict protein-protein interactions 29. RhoF-related genes were uploaded to the STRING page to determine protein interaction. Subsequently, PPI pairs were input into Cytoscape software (http://www.cytoscape.org) (version 3.7.1) to visualize and construct the PPI network 30.

GeneMANIA was used to analyze the interactions between proteins and RhoF 31.

## Results

### Comparison of the expressions of the Rho GTPase family

**Figure 2:**
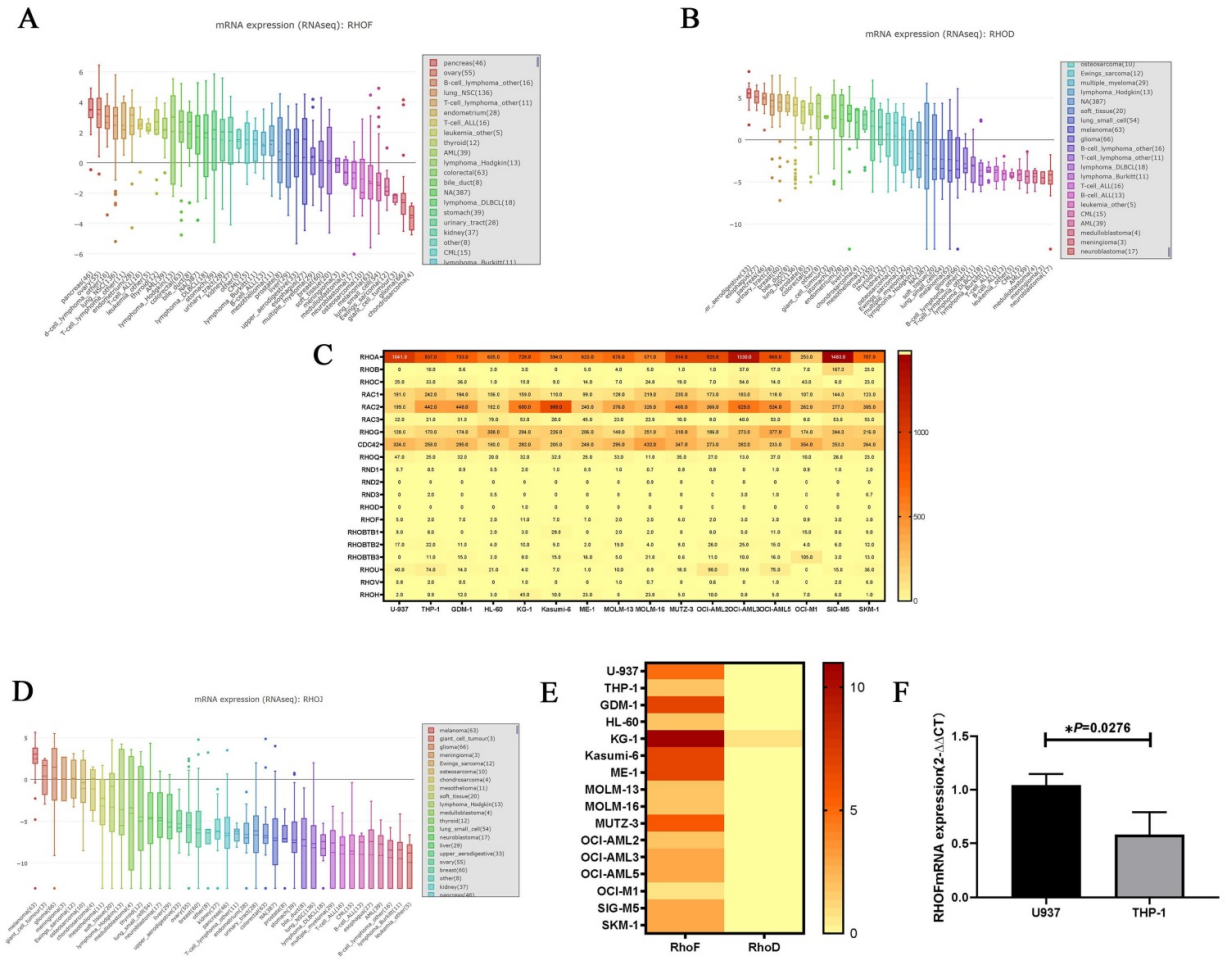
Comparison of Rho GTPase family expression levels in AML cell lines. (A, B) Expression features of RhoF and RhoD in 30 human cancer cell lines. (C) Heatmap of Rho GTPase family expression in 16 AML cell lines. (D) Expression features of RhoJ in 30 human cancer cell lines. (E) Heatmap of RhoF and RhoD expression in 16 AML cell lines. (F) Comparison of RhoF expression level in U937 and THP-1 cell lines.

The expression level of the RhoD subfamily in human cancer cell lines varied in the CCLE database. RhoF was highly expressed in hematological malignancies including lymphoma and AML (Figure 2A), whereas RhoD was expressed at a low level in leukemia (Figure 2B). The transcription level of the Rho GTPase family was detected in 16 AML cell lines using the EMBL-EBI database. As shown in Figure 2C, Rac, Cdc42, RhoF, RhoBTB, RHOU and RhoH were all well expressed in AML cells, and RhoA was the gene with the highest expression level. The expression of RhoJ was not reported in EMBL-EBI but a low level was documented in most cancer cell lines in the CCLE database (Figure 2D). The results also showed that RhoF was highly expressed in all 16 AML cell lines, especially in KG-1, whereas RhoD was not expressed in almost any of the AML cell lines (Figure 2E). Furthermore, RhoF expression in U937 cells was significantly higher than that in THP1 cells.

To verify the findings from the database, U937 and THP-1 cells were cultured and subjected to qRT-PCR to detect the mRNA expression of RhoF. As expected, the mRNA expression of RhoF in U937 cells was approximately twice as high as that of THP-1 cells (P = 0.0276) (Figure 2F).

**Figure 3:**
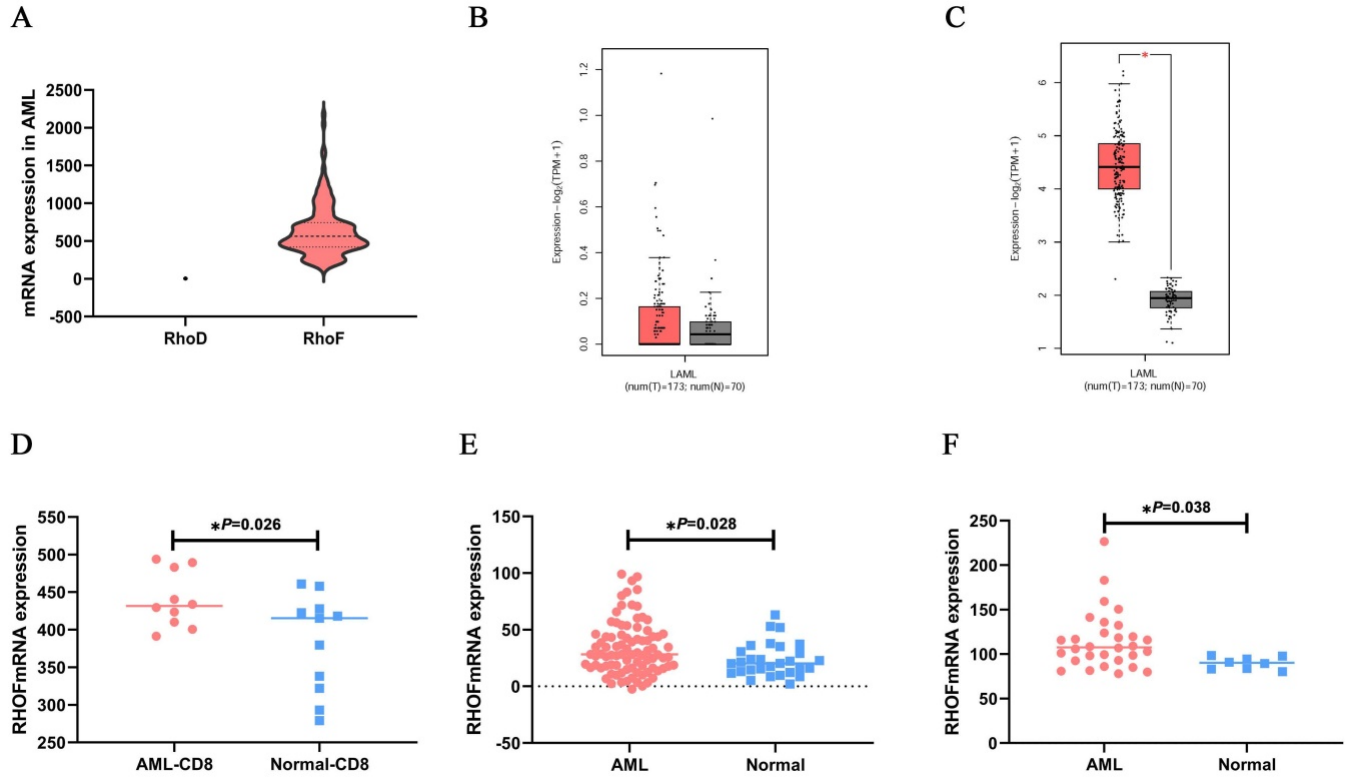
Comparison of RhoD subfamily expression levels in AML patients. (A) mRNA expression levels of RhoF and RhoD in non-M3 AML patients (n=157) from TCGA dataset (Acute Myeloid Leukemia, NEJM 2013)^24^. Expression differences of RhoD (B) and RhoF (C) between 173 de novo AML patients and 70 normal controls from GEPIA. mRNA expression levels of RhoF between AML and patients and normal samples on the GEO database series including GSE14924 (10 AML CD8+ and 11 controls, D), GSE30029 (88 AML and 29 controls, E), GSE65409 (30 AML and 8 controls, F).

Next, to determine the mRNA expression features of RhoF and RhoD in non-M3 AML patients, the RhoF and RhoD expression levels in TCGA datasets (Acute Myeloid Leukemia, NEJM 2013, n=157) 24 were compared, revealing a high RhoF expression level over that of RhoD (Figure 3A). Moreover, the results showed no significant difference in RhoD expression between AML patients (n=173) and normal controls (n=70) from GEPIA (Figure 3B). For validation, we performed a further study using a GEO microarray series to determine the expression level of RhoF. As shown in Figure 3C-F, the mRNA expression of RhoF was significantly higher than that of its normal counterparts in AML dataset from GEPIA (173 AML and 70 controls), GSE14924 (10 AML CD8+ and 11 controls), GSE30029 (88 AML and 29 controls) and GSE65409 (30 AML and 8 controls) (P<0.05 for all).

### RhoF expression and clinical characters

To further illustrate the relation between RhoF expression and clinical features, we explored the association between the mRNA expression of RhoF and clinical laboratory parameters from TCGA datasets (Acute Myeloid Leukemia, NEJM 2013)^24^. As illustrated in Table 1, RhoF was negatively correlated with the ratio of bone marrow blasts (n=157, P=0.020) and peripheral white blood cell count (n=157, P=0.003).

**Figure 4:**
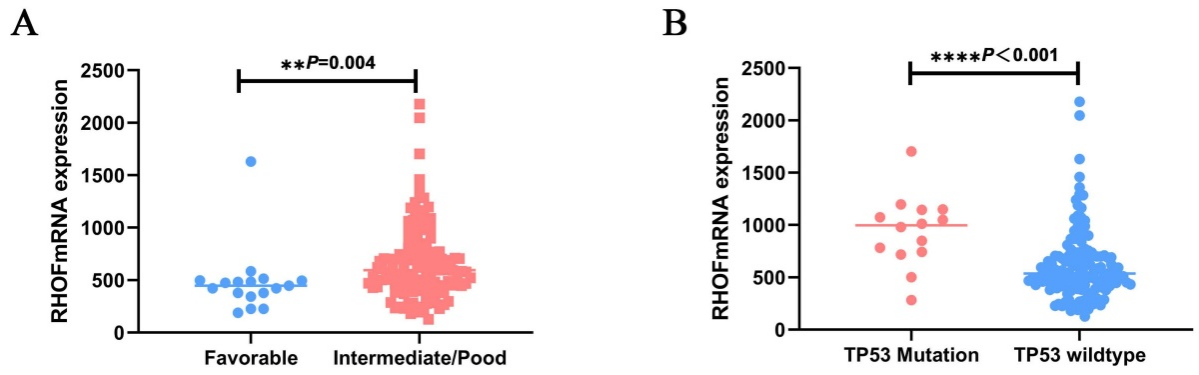
Association of RhoF expression with patient clinical features in TCGA dataset (Acute Myeloid Leukemia, NEJM 2013, n=157)^24^. Relative RhoF log2 mRNA expression in (A) patients with favorable risk stratification and intermediate/poor risk stratification; (B) patients with TP53 mutations versus wild type.

As shown in Table 2, patients with high RhoF were significantly older (52yr vs. 58yr, P = 0.013) and at a higher cytogenetic risk (P=0.001) upon diagnosis than those in the low RhoF group. The same trend can be observed in GSE12417, RhoF high group was order than low group (56yr vs. 59yr, P=0.186), but it was not statistically significant. The correlation between the cytogenetic risk classification and RhoF expression level was further determined. We found that the RhoF expression in patients with favorable and low risk was much lower than that in patients with intermediate and poor risk (P=0.001) (Figure 4A). However, no significant correlation was evident between the RhoF expression and sex, race, bone morrow blasts, white blood cell count, peripheral blood blasts, FBA type and treatment options (allo-HSCT or not) (P> 0.05 for all) (Table 2). There was also no statistical significance between FAB type and RHOF expression level in GSE12417 (P=0.648).

### RhoF and mutations

To clarify the molecular genetic aberrations that may lead to or be associated with high RhoF in AML, we explored its expression with respect to the mutational status of patients with AML. There were more IDH1 (n: 4 vs. 12, P=0.033), NRAS (n: 2 vs. 10, P=0.015) and TP53 (n: 2 vs. 12, P=0.005) mutation cases in the group with high RhoF (Table 3). Furthermore, the RhoF expression was higher in patients with TP53 mutations (n = 14) upon diagnosis than in patients with wild type TP53 (n = 143, p < 0.0001) (Figure 4B). No significant association was observed between RhoF expression and mutations in FLT3, RUNX1, IDH2, TET2, CEBPA, WT1, DNMT3A and NPM1 (Table 3).

### Rho GTPase family expression and survival in AML patients

To assess the prognostic significance of the Rho GTPase family in AML, the OS of patients with high and low expression of each Rho GTPase was compared in the TCGA dataset (Acute Myeloid Leukemia, NEJM 2013) 24 and GSE12417 (Table 4). As indicated, Rac2, Rnd2, RHOBTB1, RHOBTB3, RhoC, and RhoF were risk factors for a poor outcome only in the TCGA database (Acute Myeloid Leukemia, NEJM 2013) 24. Notwithstanding, the synthesis of the two separate databases showed that RhoF was an adverse prognostic factor and that RhoD had no significant effect on OS in AML patients.

**Figure 5:**
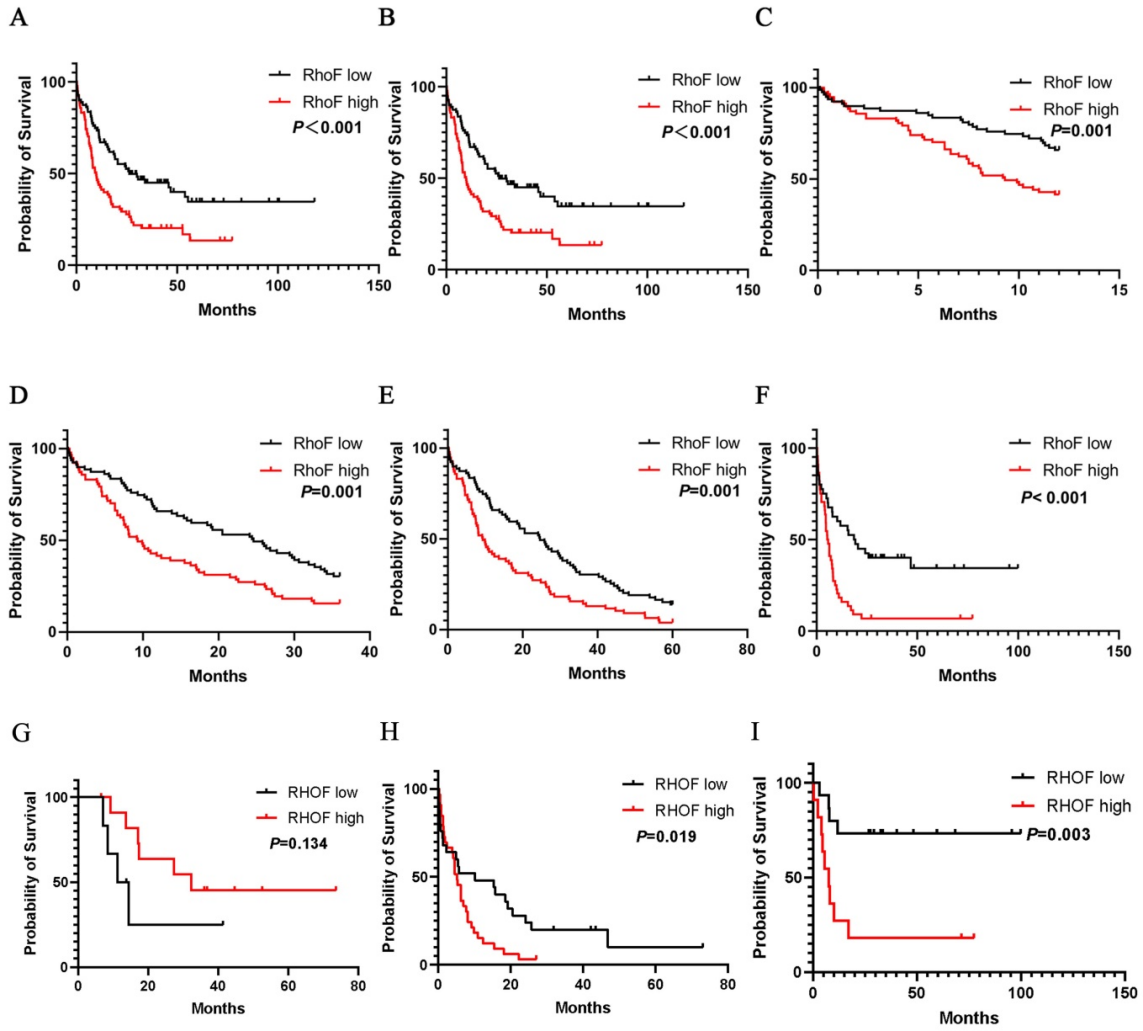
Overall survival of AML patients grouped by RhoF median cutoff in TCGA database (Acute Myeloid Leukemia, NEJM 2013, n=157) 24(A) and GSE12417 (n=162) (B). 1-year (C), 3-year (D) and 5-year (E) overall survivals comparison between high and low RhoF groups in TCGA database (Acute Myeloid Leukemia, NEJM 2013, n=157)^24^. (F) Overall survival of patients < 60 years of age with RhoF high versus RhoF low in TCGA database (Acute Myeloid Leukemia, NEJM 2013, n=80)^24^. (G) Overall survival of patients > 60 years of age receiving transplant with RhoF high versus RhoF low in TCGA database (Acute Myeloid Leukemia, NEJM 2013, n=18)^24^. (H) Overall survival of patients > 60 years of age receiving intensive chemotherapy with RhoF high versus RhoF low in TCGA database (Acute Myeloid Leukemia, NEJM 2013, n=59)^24^. (I) Overall survival of patients < 60 years of age receiving intensive chemotherapy with RhoF high versus RhoF low in TCGA database (Acute Myeloid Leukemia, NEJM 2013, n=26)^24^.

Further evaluation indicated that high RhoF expression was considerably associated with the poor overall survival of AML patients. Convincingly, the observations were validated by two independent cohorts TCGA (Acute Myeloid Leukemia, NEJM 2013) 24 and GSE12417 (Figure 5A and 5B). Moreover, a subgroup analysis of the TCGA database (Acute Myeloid Leukemia, NEJM 2013) 24 indicated that the upregulation of RhoF in AML was a risk factor for reduced 1-year (median 12.0 vs. 9.2 months, P =0.001, Figure 5C), 3-year (median 24.6 vs. 9.2 months, P=0.001, Figure 5D), and 5-year (median 24.6 vs. 9.2 months, P=0.001, Figure 5E) OS in AML patients.

When patients were stratified by their age into younger patients (< 60) and older patients (≥ 60), compared with low RhoF expression, high RhoF expression was more significantly associated with shorter OS (median 55.4 vs. 16.3 months, P=0.01, Figure 5F) in younger patients. In a more detailed exploration, the RhoF expression level was not significantly associated with OS in older patients who receive a transplant (median 18.4 vs. 44.1 months, P=0.134, Figure 5G). However, high expression of RhoF in older patients receiving intensive chemotherapy suggested a poor prognosis (median 24.1 vs. 8.2 months, P=0.019, Figure 5H), which was analogous to the trend in younger patients (median 75.3 vs. 19.5 months, P=0.003, Figure 5I).

**Figure 6:**
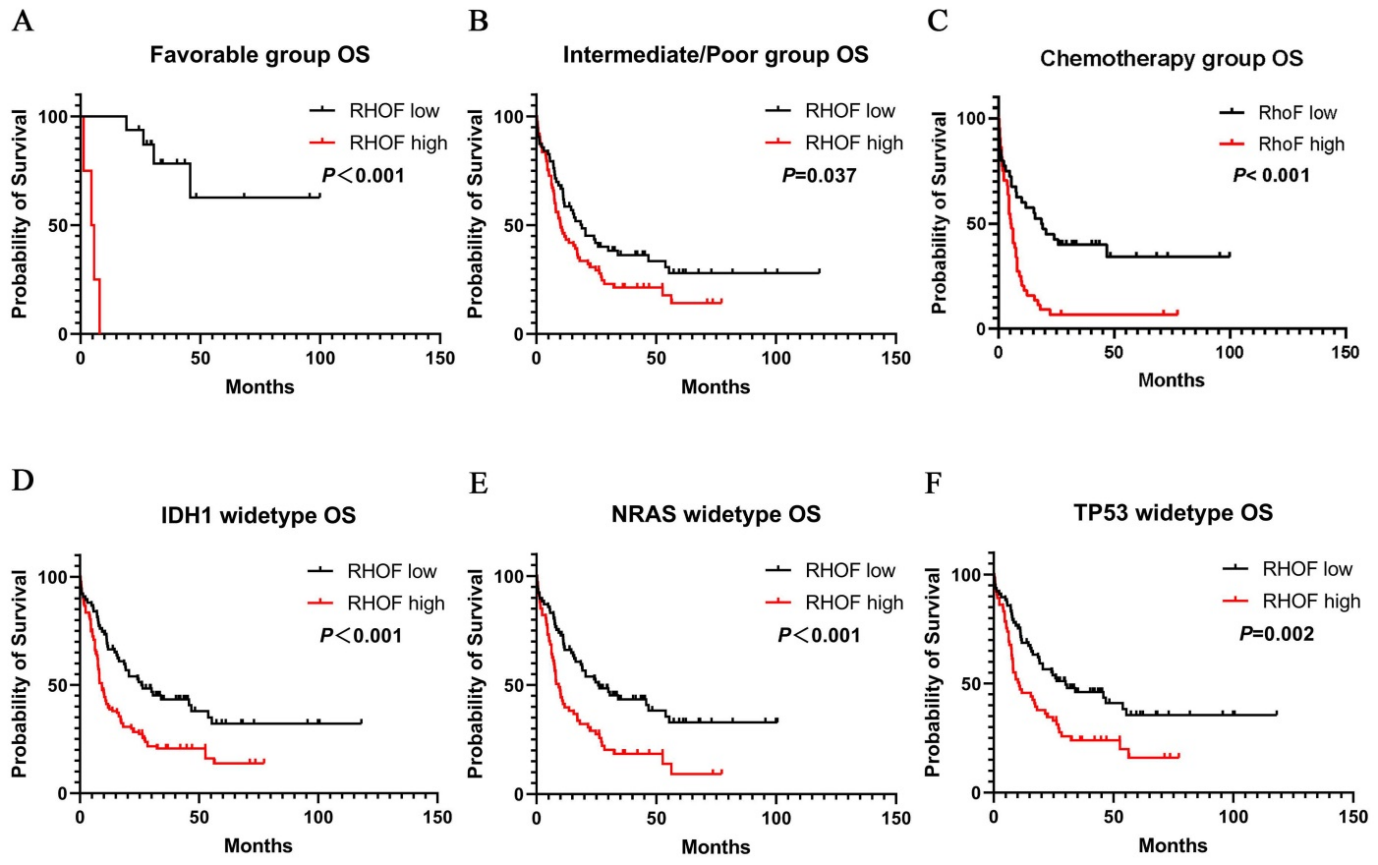
Survival analysis of patients with respect to RhoF expression in TCGA database (Acute Myeloid Leukemia, NEJM 2013, n=157)^24^. (A) Overall survival of patients with RhoF high versus RhoF low in patients with favorable risk stratification (n=20). (B) Overall survival of patients with RhoF high versus RhoF low in patients with intermediated/poor risk stratification (n=137). (C) Overall survival of patients with RhoF high versus RhoF low in patients without transplant (n=85). Overall survival of patients with RhoF high versus RhoF low among patients with IDH1 (n=141) (D), NRAS (n=145) (E) and TP53 (n=143) (F) wild-type gene.

A similar prognostic impact of RhoF expression was also present in AML favorable patients (median 33.5 vs. 4.5 months, P<0.001, Figure 6A) and intermediate/poor patients (median 19.0 vs. 10.0 months, P=0.02, Figure 6B) with AML stratified according to their risk. Immediately afterwards, we screened patients who received intensive chemotherapy and patients who received intensive chemotherapy followed by allo-SCT in the cohort to analyze the relationship between RhoF expression and survival. High RhoF levels significantly contributed to worse OS in AML patients who received intensive chemotherapy (median 18.5 vs. 5.3 months, P<0.001, Figure 6C). A similar but no statistically significant trend in OS was observed in patients who received intensive chemotherapy followed by allo-HSCT.

Additionally, we examined the association between RhoF expression and clinical outcome in patients with IDH1, NRAS and TP53 mutations. As expected, the upregulation of RhoF was associated with a significantly shorter OS in patients with wild type IDH1 (median 25.8 vs. 8.1 months, P<0.001, Figure 6D), NRAS (median 25.8 vs. 8.2 months, P<0.001, Figure 6E) and TP53 (median 30.0 vs. 10.2 months, P=0.02, Figure 6F).

In the multivariate Cox survival analysis, high RhoF expression associated with shorter OS was not detected after adjusting for age, cytogenetic risk, allo-SCT status, and TP53 mutation (HR =1.464, 95% CI: 0.961-2.232, P =0.076) (Table 5). Next, we performed a multivariate Cox analysis of RhoF expression in AML patients who received intensive chemotherapy, and found that a high level of RhoF expression was an independent risk factor for worse OS (HR =1.770, 95% CI: 1.013-3.092, P =0.045) (Table 6).

### KEGG, GO and GSEA enrichment analysis

**Figure 7:**
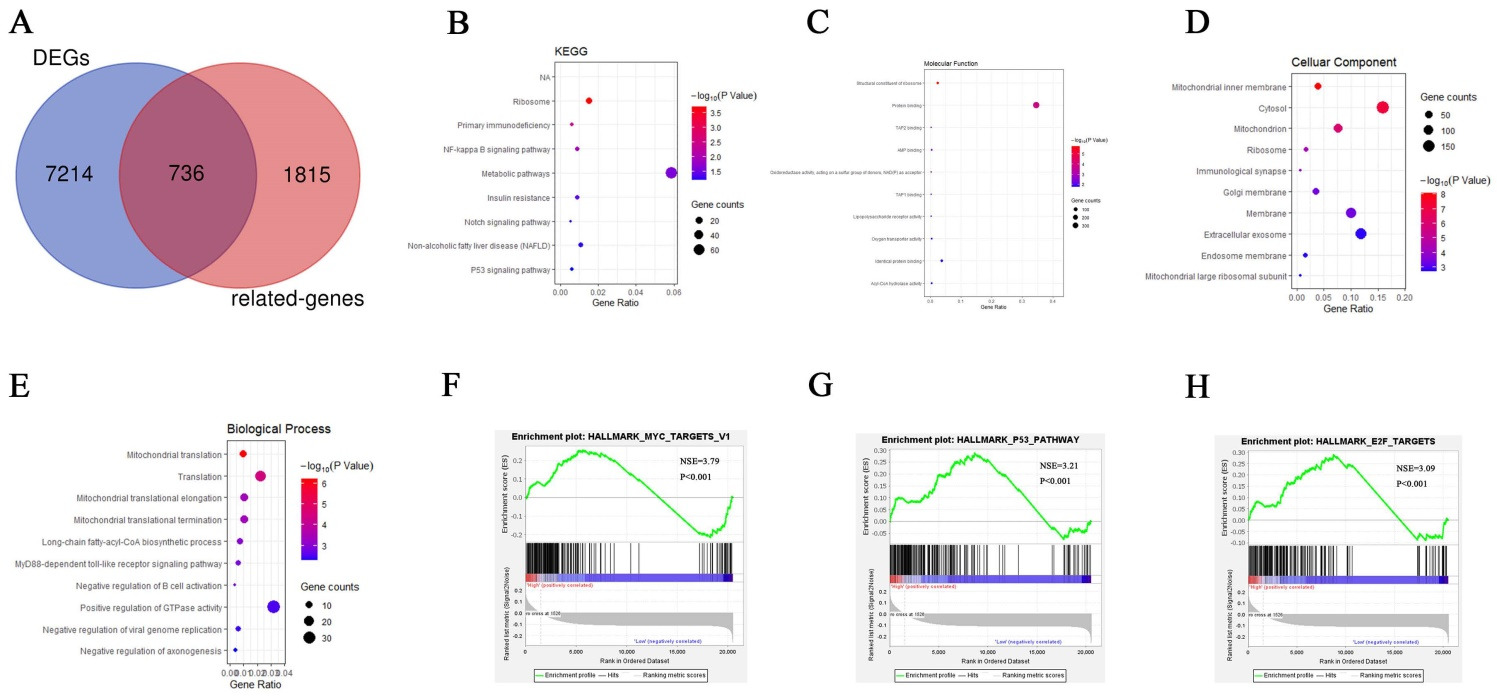
Venn diagram showed 736 RhoF-related genes (A). KEGG and GO biological function enrichment analyses of 736 RhoF related genes. KEGG signal pathway enrichment analysis (B); Molecular function enrichment analysis (C), Cell component enrichment analysis (D) and biological process enrichment analysis (E). GSEA analysis of TCGA dataset (Acute Myeloid Leukemia, NEJM 2013, n=157)^24^ based on RhoF expression, and MYC targets (F), P53 pathways (G) and E2F targets (H) were screened out.

The KEGG pathway enrichment analysis of 736 RhoF-related genes screened by Venn diagram (Figure 7A) showed that the most enriched pathways included ribosome, primary immunodeficiency, NF-kappa B pathway, metabolic pathway, insulin resistance, notch signaling, nonalcoholic fatty liver disease (NAFLD) and P53 signaling pathway (Figure 7B). GO biological process enrichment analysis revealed that the involved processes were the structural constituent of ribosome at the MF levels, mitochondrial inner membrane at the CC levels, and mitochondrial translation at the BP levels (Figure 7C-E).

To gain further biological insights into the underlying mechanisms of RhoF overexpression in AML, GSEA analysis was performed and revealed that the gene sets were significantly enriched in MYC targets (NES =3.79, P <0.001), P53 pathways (NES =3.21, P<0.001) and E2F targets (NES =3.09, P<0.001) (Figure 7F-H).

### PPI analysis

**Figure 8:**
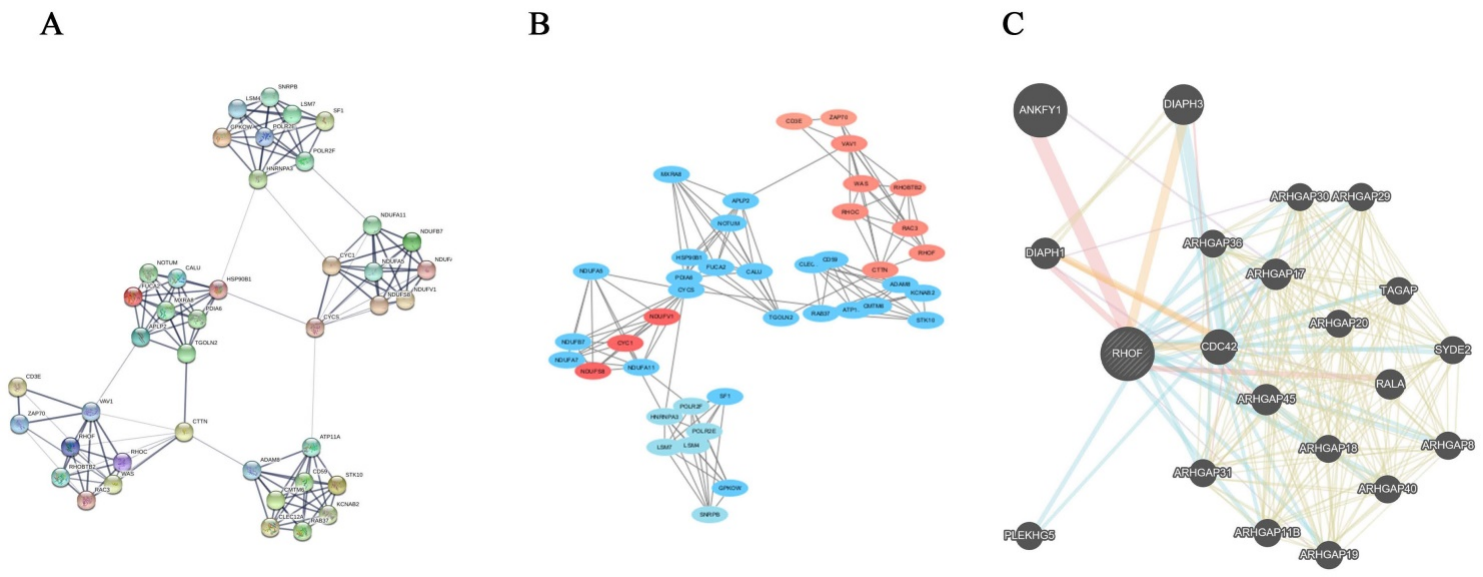
The PPI analysis of RhoF and RhoF-related genes. Genes from catalytic activity pathway by STRING online tool (A) and Cytoscape software (B). Protein-protein interaction network of RhoF analyzed by GeneMANIA (C).

The RhoF-related genes were uploaded to the STRING website for PPI analysis (Figure 8A). The most significant pathway was identified using the MCODE application from Cytoscape software (Figure 8B). Additionally, the protein-protein interactions of RhoF were determined using GeneMANIA online tools. The results showed that ANKFY1, Cdc42 and DIAPH3 interacted with RhoF (Figure 8C).

### Gene Coexpression Network Analysis

**Figure 9:**
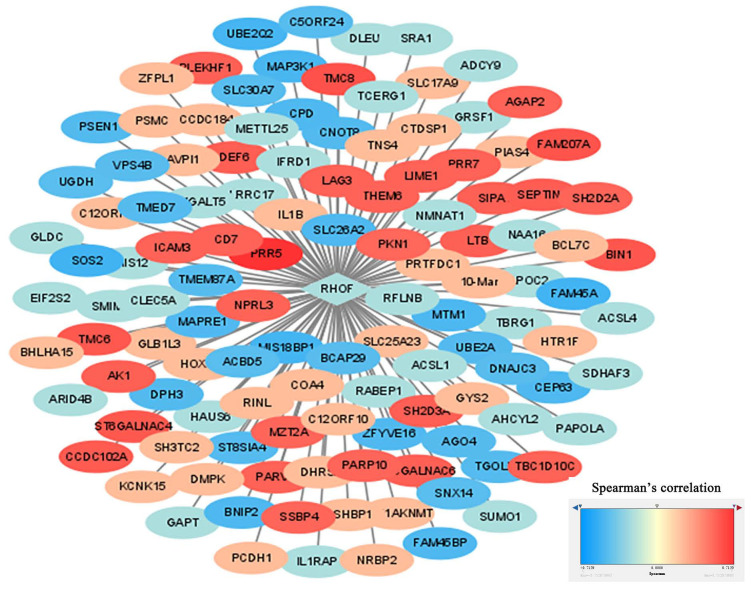
Construction of RhoF co-expressed genes networks by Cytoscape software. Blue represents top 50 genes that are negatively related to RhoF, and red points out top 50 genes that are positively related to RhoF. The darker the color, the stronger the correlation.

The identification of RhoF-coexpressed genes was completed with the cBioPortal dataset online tool. The top 10 positive genes were PRR5, TMC8, FAM207A, BIN1, TMC6, ST6GALNAC4, CCDC102A, SEPTIN1, DEF6 and SH2D3A. The top 10 negative genes were SOS2, FAM45A, MIS18BP1, UBE2Q2, MAP3K1, MTM1, SLC26A2, CEP63, FAM45BP and BNIP2. The process was visualized via Cytoscape (version 3.7.1) (Figure 9).

## Discussion

As a new research focus, the small Rho GTPase in cancers has received increasing attention over the past years, but its role in leukemia has barely been elucidated. Studies have reported that the activation of the RhoA/ROCK1/PTEN pathway induces the proliferation of human leukemia cells in a mouse leukemia xenograft model 32 and that the Ras-MAPK and RhoA signaling pathways may result in proliferation, survival time extension and angiogenesis induction of AML cells 33. Due to SMARCB1 deficiency, GEFs can cause Rac GTPase activation and increase AML cell migration and survival 34. Increased expression and activity of Cdc42 are associated with the transformation of HSCs/P to AML in leukemia cells 13. In addition, two independent studies of the atypical Rho GTPase family have demonstrated that the low expression of the RhoH transcript is a predictor of worse prognosis in AML and ALL 35, 36. Given these findings, the role of the RhoD subfamily in AML remains obscured. To the best of our knowledge, the current study is the first to report the clinical implications of RhoD subfamily expression in AML. The RhoF gene seems to be a special member because of its elevated expression and adverse prognostic impact in AML.

Our results showed for the first time that the expression levels of RhoF in CD8+ T cells (GSE14924), CD34+ or CD34- bone marrow cells (GSE30029, TCGA) and peripheral blood mononuclear cells (PBMCs) (GSE65409) in AML patients were obviously higher than those in their normal counterparts. In 16 AML cell lines, Rac, Cdc42, RhoF, RhoBTB, RHOU and RhoH were all well expressed, and RHOA was the gene with the highest expression level. However, as the other member of the RhoD subfamily, RhoD had a low expression level in AML and produced no statistically significant effect on the prognosis, which has rarely been studied in other cancers. Comparisons of the prognostic significance of Rho GTPase family expression were performed among datasets from both cytogenetically normal (GSE12417) and cytogenetically heterogeneous AML patients (TCGA). Rac2, Rnd2, RHOBTB1, RHOBTB3, RHOC and RHOF were risk factors for a poor outcome only in the TCGA database, although RhoF was the only gene with survival significance in two independent cohorts. The overexpression of RhoF led to poor prognosis in both good cytogenetic risk and poorer cytogenetic risk groups. Furthermore, the upregulation of RhoF expression was also a risk factor for declining 1-, 3-, and 5-year survival rates in AML patients. These findings evidence that high RhoF expression is an adverse factor in AML overall survival. We hypothesize that the high RhoF expression reinforces its potential as a powerful and simple prognostic marker that seems to be independent of cytogenetic abnormalities, which would await further confirmation.

Moreover, we observed that RhoF was an independent risk factor in AML patients who underwent chemotherapy alone, but not in patients who also underwent allo-HSCT, suggesting that the unfavorable effect of RhoF overexpression might be overcome by allo-HSCT. Although RhoF expression is strongly associated with older age, the upregulation of RhoF expression may affect the prognosis of younger patients, resulting in the poor OS. The significantly worse OS of patients with high RhoF expression was also related to wild type TP53, IDH1, and NRAS. Thus, our data emphasize that high RhoF expression has a significant impact on prognosis in younger adults with AML without transplantation and mutation.

Furthermore, a higher RhoF expression was closely associated with special clinical features, including older age, intermediate-/poor-risk cytogenetics and mutations in IDH1, NRAS, and TP53. Contrary to expectations, the transcription level of RhoF was negatively correlated with the proportion of BM blasts and WBCs. However, RhoF was enriched in AML bone marrow cells and PBMCs, which is evident in more than one data set. Because no published articles have reported on this aspect, further work is needed to investigate the potential correlation between RhoF and BM blasts and WBCs.

Previous work on RhoF focused on its structural and functional characterization. Our biological function analysis strongly speculates that RhoF has untapped potential in the oncogenesis of AML. The KEGG and GO results suggest that RhoF-related genes participate in the NF-kappa B pathway, notch signaling, P53 signaling pathway and mitochondrial translation. As the NF-kappa B and P53 signaling pathways have been demonstrated to be molecular pathogenesis and therapeutic targets of AML 37, 38 and RhoF can be activated by KLF4, including NF-κB signaling in esophageal keratinocytes 39, it follows that activated RhoF may have the same effect in AML by activating the NF-kappa B. High levels of notch can interfere with the drug response, which can be used as a prognostic marker and therapeutic target in AML 40. These findings signify that RhoF may have a role in the occurrence of AML through the above channels. Meanwhile, GSEA analysis assumes that high RhoF expression is involved in several epigenetic regulation gene sets in AML patients, such as MYC, which provides a potential direction for further exploration of its biological functions.

PPI analysis suggests that the RhoF protein interacts with other Rho GTPases to increase its catalytic activity to perform its biological and chemical functions. ANKFY1, Cdc42 and DIAPH3 directly interact with RhoF from GeneMANIA. Cdc42 promotes the occurrence of AML. DIAPH3 has been identified as the binding protein of STK38 that impairs the interaction between STK38 and MEKK and activates ERK signaling to trigger off tumorigenesis of lung cancer 41. These findings may explain why RhoF may act as a tumor suppressor in AML.

In addition, the RhoF co-expression network suggests that RhoF may have a synergistic effect on AML with other oncogenic signaling pathways, which also indicates its potential molecular mechanism in tumorigenesis. Therefore, we can boldly speculate that tumor suppressor genes antagonize the tumor-promoting effect of RhoF.

In summary, RhoF, which is aberrantly expressed in AML patients and AML cell lines, is the only member of the RhoD subfamily that has prognostic significance in non-M3 AML. Its overexpression is an unfavorable prognostic marker for non-M3 AML. Notably, RhoF is an independent poor survival factor for patients receiving intensive chemotherapy and a potential therapeutic target for the treatment of AML. A multitude of prospective studies are necessary to support our observations, such as a large number of clinical samples to verify the expression level and the impact on survival, in vivo and in vitro experiments to gain insights into its function of enhancing the proliferation of AML cells. Moreover, further mechanistic studies are needed to delineate how RhoF participates in the biology of the hematopoietic system and its function in modulating unfavorable prognostic impacts in AML.

## Figures and Tables

**Table 1:** Correlation between RhoF expression and clinical and laboratorial parameters in TCGA dataset (Acute Myeloid Leukemia, NEJM 2013) 24.

	RhoF (n=157)	
	r	p
Age	0.155	0.053
BM blasts	-0.186	0.020
WBC	-0.239	0.003
PB blasts	-0.035	0.662

**Abbreviations:** WBC: white blood cell; BM: bone marrow; PB: peripheral blood.

**Table 2:** Characteristics of AML patients between RhoF high and low groups in TCGA dataset (Acute Myeloid Leukemia, NEJM 2013) 24.

	Total	RhoF low (n=79)	RhoF high (n=78)	P
Sex, n (%)				0.941
Male	85	43(54.4)	42(53.8)	
Female	72	36(45.6)	36(46.2)	
Race, n (%)				0.794
White	115	58(73.2)	57(73.1)	
Black	6	3(3.8)	3(3.8)	
Asian	1	1(1.3)	0	
Other	35	17(21.5)	18(23.1)	
FAB, n				0.167
M0	16	6	10	
M1	44	19	25	
M2	38	21	17	
M4	34	22	12	
M5	18	10	8	
M6	2	0	2	
M7	3	0	3	
NA	2	1	1	
Age, n				0.013
<60	80	48	32	
≥60	77	31	46	
BM blasts, %				0.609
Median(range)		81.54 (32-100)	76.43 (30-99)	
WBC(×109/L)				0.350
Median(range)		87.88 (1-297.4)	70.01 (0.6-171.9)	
PB blasts, %				0.737
Median(range)		79.79(0-98)	77.21(0-97)	
NCCN, n (%)				0.001
Favorable	17	15(18.75)	2(2.6)	
Intermediate	92	48(60)	44(57.1)	
Poor	45	15(18.75)	30(39.0)	
NA	3	2(2.5)	1(1.3)	
Allo-SCT, n (%)				0.375
Yes	72	39(24.8)	33(21.0)	
No	85	40(25.5)	45(28.7)	

**Abbreviations:** FAB: French American British. NA: Not Applicable.

**Table 3:** Mutation status of AML patients between RhoF high and low groups in TCGA dataset (Acute Myeloid Leukemia, NEJM 2013) 24.

	Total	RhoF low (n=79)	RhoF high (n=78)	P
FLT3, n (%)				0.685
Mut	44(28)	21(26.5)	23(29.5)	
Wt	113(72)	58(73.5)	55(70.5)	
IDH1, n (%)				0.033
Mut	16(10.2)	4(5)	12(15.4)	
Wt	141(89.8)	75(95)	66(84.6)	
IDH2, n (%)				0.458
Mut	17(10.8)	10(12.7)	7(9)	
Wt	140(89.2)	69(97.3)	71(91)	
RUNX1, n (%)				0.806
Mut	15(9.6)	8(10.1)	7(9)	
Wt	142(90.4)	71(89.9)	71(91)	
TET2, n (%)				0.806
Mut	15(9.6)	8(10.1)	7(9)	
Wt	142(90.4)	71(89.9)	71(91)	
NRAS, n (%)				0.015
Mut	12(7.6)	2(2.5)	10(12.8)	
Wt	145(92.4)	77(97.5)	68(87.2)	
CEBPA, n (%)				0.398
Mut	13(8.3)	8(10.1)	5(6.4)	
Wt	144(91.7)	71(89.9)	73(93.6)	
WT1, n (%)				0.527
Mut	10(6.4)	6(7.6)	4(5.1)	
Wt	147(93.6)	73(92.4)	74(94.9)	
DNMT3A, n (%)				0.092
Mut	41(26.1)	16(20.3)	25(32.1)	
Wt	116(73.9)	63(79.7)	53(67.9)	
NPM1, n (%)				0.958
Mut	48(30.6)	24(30.4)	24(30.8)	
Wt	109(69.4)	55(69.6)	54(69.2)	
TP53, n (%)				0.005
Mut	14(8.9)	2(2.5)	12(15.4)	
Wt	143(91.1)	77(97.5)	66(84.6)	

**Table 4:** Comparison of Overall Survival between different expression levels of Rho GTPase family in TCGA datasets (Acute Myeloid Leukemia, NEJM 2013)^24^ and GSE12417.

Variables	TCGA (n=157)			GSE12417 (n=162)	
	χ2	P	χ2		P
RhoF	13.291	<0.001	4.143		0.042
RhoD	0.08	0.777	3.394		0.065
RhoA	0.042	0.838	0.324		0.569
RhoB	0.096	0.757	0.033		0.857
RhoC	6.602	0.010	2.761		0.097
Rac1	0.000	0.996	0.060		0.806
Rac2	4.211	0.040	0.030		0.862
Rac3	0.804	0.370	0.169		0.681
RhoG	0.006	0.940	0.790		0.374
Cdc42	0.026	0.871	0.139		0.709
RhoQ	3.068	0.080	0.270		0.603
Rnd1	3.694	0.055	0.073		0.787
Rnd2	6.157	0.013	0.034		0.853
Rnd3	0.348	0.555	0.018		0.894
RhoBTB1	4.378	0.036	0.229		0.632
RhoBTB2	3.523	0.061	1.630		0.202
RhoBTB3	12.237	<0.001	0.242		0.623
RhoU	0.200	0.655	0.924		0.336
RhoV	0.924	0.336	0.013		0.908
RhoH	0.133	0.715	0.388		0.534
					

**Note:** GSE12417: GEO microarray series

**Table 5:** Multivariate Analysis (Cox regression) on the Overall Survival in AML patients from TCGA datasets (Acute Myeloid Leukemia, NEJM 2013) 24.

Variables	OS (n=157)	
	HR (95% CI)	p
Age (< 60 v. ≥ 60 years)	1.534 (0.981-2.398)	0.061
Cytogenetic risk (favorable vs. intermediate/poor)	3.094 (1.283-7.464)	0.012
Allo-HSCT (yes vs. no)	0.413 (0.268-0.636)	0.000
TP53 (WT vs. mutated)	2.304(1.231-4.315)	0.009
RhoF (high vs. low)	1.464 (0.961-2.232)	0.076

**Table 6:** Multivariate Analysis (Cox regression) on the Overall Survival in AML patients from TCGA datasets (Acute Myeloid Leukemia, NEJM 2013) 24 who received intensive chemotherapy.

Variables	OS (n=85)	
	HR (95% CI)	p
Age (< 60 v. ≥ 60 years)	2.037(1.002-4.143)	0.049
RhoF (high vs. low)	1.770(1.013-3.092)	0.045
TP53 (WT vs. mutated)	1.979(0.953-4.109)	0.067
Cytogenetic risk (favorable vs. intermediate/poor)	3.730(1.063-13.083)	0.040
IDH1(WT vs. mutated)	1.318(0.437-3.972)	0.624
NRAS (WT vs. mutated)	0.488(0.187-1.278)	0.144
