# High Expression of RhoF Predicts Worse Overall Survival: A Potential Therapeutic Target for non-M3 Acute Myeloid Leukemia: Erratum

**DOI:** 10.7150/jca.85287

**Published:** 2023-04-29

**Authors:** Yue Hou, Jie Zi, Zheng Ge

**Affiliations:** Department of Hematology, Zhongda Hospital, Medical School of Southeast University, Institute of Hematology Southeast University, Nanjing 210009, China

In the initially published version of our article, we found that there are the errors in Figure 5. Specifically, the survival curve of Figure 5B and 5F are incorrect. The errors occurred during assembling the figures. The correct Figure 5 is provided below. This correction will not affect the results and conclusions. The authors apologize for any inconvenience this may cause.

## Figures and Tables

**Figure 5:**
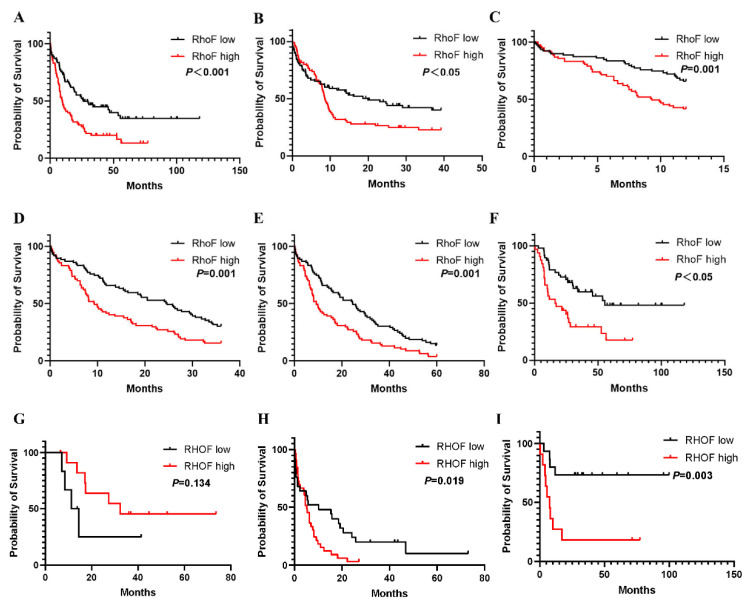
Overall survival of AML patients grouped by RhoF median cutoff in TCGA database (Acute Myeloid Leukemia, NEJM 2013, n=157) [24](A) and GSE12417 (n=162) (B). 1-year (C), 3-year (D) and 5-year (E) overall survivals comparison between high and low RhoF groups in TCGA database (Acute Myeloid Leukemia, NEJM 2013, n=157)[24]. (F) Overall survival of patients < 60 years of age with RhoF high versus RhoF low in TCGA database (Acute Myeloid Leukemia, NEJM 2013, n=80)[24]. (G) Overall survival of patients > 60 years of age receiving transplant with RhoF high versus RhoF low in TCGA database (Acute Myeloid Leukemia, NEJM 2013, n=18)[24]. (H) Overall survival of patients > 60 years of age receiving intensive chemotherapy with RhoF high versus RhoF low in TCGA database (Acute Myeloid Leukemia, NEJM 2013, n=59)[24]. (I) Overall survival of patients < 60 years of age receiving intensive chemotherapy with RhoF high versus RhoF low in TCGA database (Acute Myeloid Leukemia, NEJM 2013, n=26)[24].

